# Flow cytometric characterisation of the complex polyploid genome of *Saccharum officinarum* and modern sugarcane cultivars

**DOI:** 10.1038/s41598-019-55652-3

**Published:** 2019-12-18

**Authors:** Cushla J. Metcalfe, Jingchuan Li, Debora Giorgi, Jaroslav Doležel, Nathalie Piperidis, Karen S. Aitken

**Affiliations:** 1 CSIRO Agriculture and Food, Queensland Biosciences Precinct, 306 Carmody Rd, St Lucia, QLD 4067 Australia; 2 ENEA, CASACCIA Research Center, Biotech Laboratory, Via Anguillarese 301, 00123 Rome, Italy; 3 Institute of Experimental Botany, Czech Academy of Sciences, Centre of the Region Hana for Biotechnological and Agricultural Research, Šlechtitelů 31, 779 00 Olomouc, Czech Republic; 4 SRA Limited, Mackay, QLD Australia

**Keywords:** Flow cytometry, Plant evolution, Cytogenetics, Polyploidy in plants

## Abstract

Sugarcane (*Saccharum* spp.) is a globally important crop for sugar and bioenergy production. Its highly polyploid, complex genome has hindered progress in understanding its molecular structure. Flow cytometric sorting and analysis has been used in other important crops with large genomes to dissect the genome into component chromosomes. Here we present for the first time a method to prepare suspensions of intact sugarcane chromosomes for flow cytometric analysis and sorting. Flow karyotypes were generated for two *S. officinarum* and three hybrid cultivars. Five main peaks were identified and each genotype had a distinct flow karyotype profile. The flow karyotypes of *S. officinarum* were sharper and with more discrete peaks than the hybrids, this difference is probably due to the double genome structure of the hybrids. Simple Sequence Repeat (SSR) markers were used to determine that at least one allelic copy of each of the 10 basic chromosomes could be found in each peak for every genotype, except R570, suggesting that the peaks may represent ancestral *Saccharum* sub genomes. The ability to flow sort *Saccharum* chromosomes will allow us to isolate and analyse chromosomes of interest and further examine the structure and evolution of the sugarcane genome.

## Introduction

Sugarcane (*Saccharum*) is the world’s main sugar-producing crop, it currently accounts for about 80% of global sugar production^1^ and by 2022 is projected to supply 40% of the world’s first-generation biofuels^2^. In 2017 it was ranked the world’s largest crop by tonnes harvested and eighth by value with a gross productive value of USD $87 billion^3^. It is a member of the family Poaceae (the grasses), which also includes most of the world’s food and feed crops, such as rice and wheat^4^. A n = 5 is considered the ancestral haploid chromosome number for the grasses^5^, although a n = 7 has also been proposed^6^. In the scenario with an ancestral karyotype of n = 5, a whole genome duplication (WGD), chromosomal translocations and fusions resulted in an n = 12 karyotype^7^. This was followed by lineage specific and family specific shuffling, resulting in the karyotypes seen in rice, wheat, maize and sorghum. The common ancestor of the *Saccharum* is most likely a diploid with n = 10, as in sorghum^8^. The high chromosome numbers in sugarcane suggest that there were at least two further WGDs in the *Saccharum* lineage^9^. Within *Saccharum* there are two main lineages, one with *S. spontaneum* (2n = 40–128), a wild species with good general vigour and adaptation to a range of environmental stresses, and one with all other *Saccharum* species, including *S. officinarum* (2n = 80), which is the domesticated high sugar species^10^.

Modern sugarcane cultivars are derived from crosses between *S. officinarum* and *S. spontaneum* initially made by early sugarcane breeders in Java and India at the end of the nineteenth century^11^. F_1_ hybrids were backcrossed to *S. officinarum* in a process known as ‘nobilisation’. Hybrids between *S. officinarum* and *S. spontaneum* show a 2n + n transmission, where 2n is the entire chromosome set of *S. officinarum*. Early breeders used this phenomenon to introduce vigour and resistance from *S. spontaneum* while quickly recovering the high sugar content of *S. officinarum*^9^. Because the two species have different basic chromosome numbers with *S. officinarum* (x = 10) and *S. spontaneum* (x = 8), the resultant hybrid cultivars are polyploid and aneuploid with 100 to 120 chromosomes^12^. The reduction in the basic chromosome number from 10 to 8 involved two rearrangements each involving 3 sets of ancestral chromosomes^13^. This resulted in cultivars with a complex set of chromosomes with approximately 80–90% inherited from *S. officinarum*, 10–20% from *S. spontaneum* and a small percentage of recombinant chromosomes^14,15^.

The complex polyploid nature of the sugarcane genome, along with the large number of chromosomes, and the high representation of repetitive and transposable elements it shares with other plant genomes^16^, has hindered progress in understanding the genome structure. An approach that has been successfully used in many plants species is to break down the complexity of the genome by using flow cytometric sorting to isolate chromosomes or groups of chromosomes according to their relative DNA content^17^. Flow cytometry analysis of chromosomes is based on the measurement of the intensity of fluorescence of a single chromosome as it passes through an intense and focused light beam. The intensity of fluorescence is therefore directly correlated with the chromosome size^18^.

Generating the flow karyotype for sugarcane required optimisation of the method of Vrána *et al*.^19^. Of the 25 species for which we could find published protocols, almost all are grown from seed, and most are also temperate inbred species. Sugarcane is highly heterozygous so flow sorting chromosomes isolated from seeds is not possible. Sugarcane is also propagated from setts, that is, sections of stalk containing a single bud. These setts on germination generate adventitious roots which, although relatively uneven in growth, are fast growing and therefore can be used for synchronisation of the cell cycle to isolate condensed mitotic chromosomes. Sugarcane is also a tropical plant, so particular care had to be taken to keep all solutions and materials at a suitable temperature.

Here we have successfully modified the protocol of Vrána *et al*.^19^ to classify isolated mitotic metaphase chromosomes according to their relative DNA content and generate the first flow karyotypes for sugarcane. We generated flow karyotypes for two *S. officinarum* genotypes, Badila and Comus, and three hybrid cultivars, an early hybrid, Nco310, and two modern hybrid cultivars, Q165 and R570. For each genotype, we isolated, purified and amplified groups of chromosomes based on their relative DNA content. Chromosome specific Simple Sequence Repeat (SSR) markers were used to examine the chromosomal component of each peak.

The ability to flow sort sugarcane chromosomes and generate flow karyotypes will allow us to further examine the structure and evolution of the sugarcane genome and to isolate a chromosome or chromosomes of interest. The isolation of chromosomes makes it possible to, for example, analyse chromosomes with genes of interest, such as those associated with disease or pest resistance. It could also be used to sequence single chromosomes as part of a whole genome sequencing strategy. Finally, isolation and sequencing of homo(eo)logous chromosomes could be used to examine synteny between and the structure of homo(eo)logous chromosomes.

## Results

The procedure for flow cytometric analysis and sorting of plant chromosomes can be broken down into the following steps: 1. induction of cell cycle synchrony and accumulation of cells in metaphase 2. preparation of suspensions of intact chromosomes 3. flow karyotyping and sorting and 4. processing of flow-sorted chromosomes^19^.

Synchronisation of the cells depends on a 2-step cell-cycle process. Cells are arrested and blocked at the interface between G1 and early S phase of cell cycle, usually with hydroxyurea (HU)^20^. Upon the release from the block, the cells traverse S and G_2_ phases and enter mitosis in a synchronous manner. Mitotic cells are then accumulated at metaphase using a mitotic spindle inhibitor, most commonly amiprophos methyl (APM). Oryzalin, colchicine, trifluralin and N_2_O under pressure can also be used in place of APM^19^, however, APM is relatively cheap, more readily available and easier to use. For each species the following parameters need to be optimised, HU concentration, the recovery time (the time between the release from the HU block and treatment in the mitotic spindle inhibitor), mitotic spindle inhibitor concentration and treatment time.

### Cell cycle synchronisation and chromosome isolation

**Figure 1:**
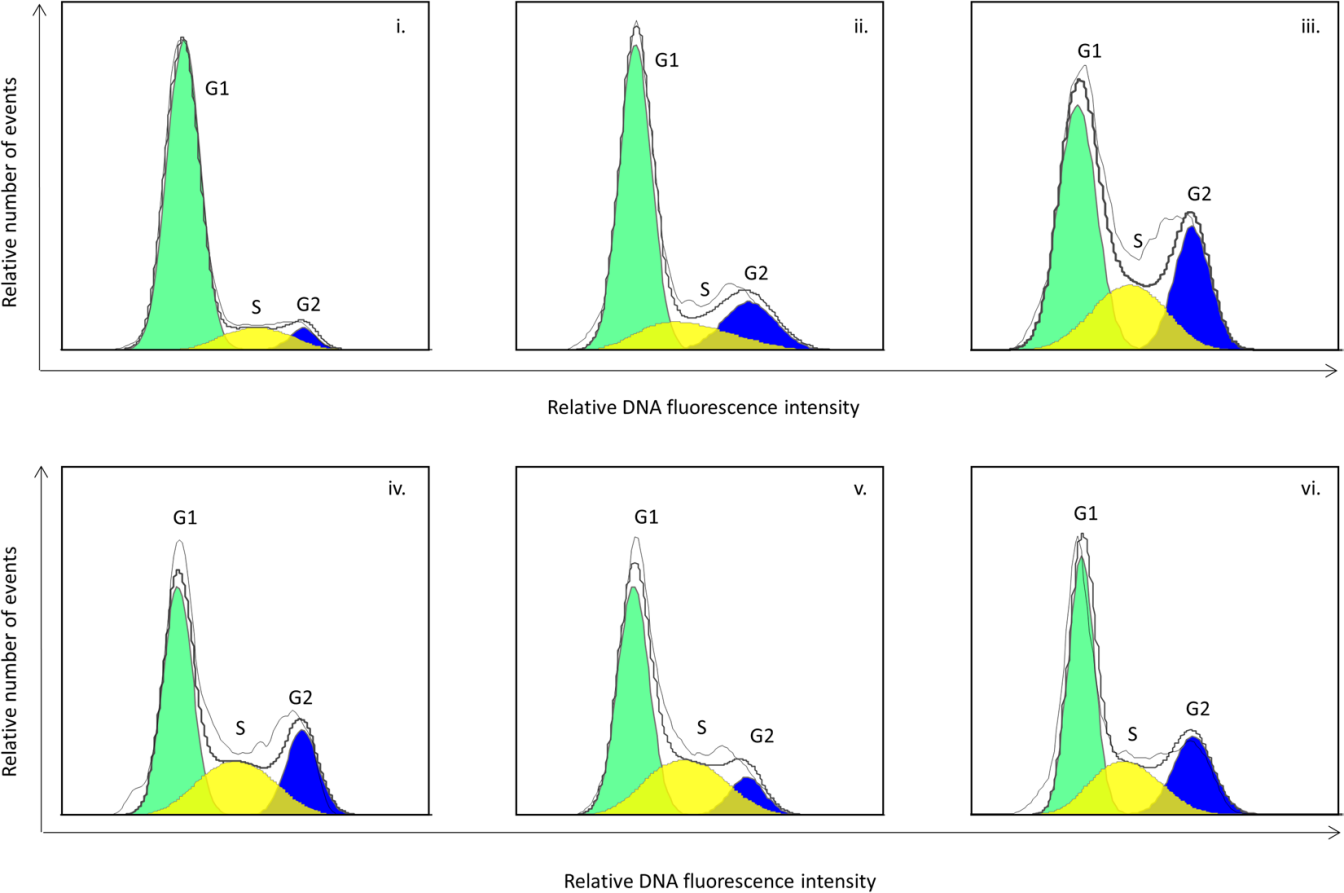
The effect of hydroxyurea (HU) at five concentrations on cell cycle of sugarcane root tip cells. Setts were treated in HU for 18 hours, root tips were excised and nuclei were isolated for flow cytometric analysis of DNA content immediately after the setts were removed from the HU treatment. Histograms show the relative DNA content (linear scale) of nuclei in G1 (green), S (yellow) and G2 (blue) cell cycle phases. (i) untreated, (ii) 2.0 mM HU, (iii) 2.5 mM HU, (iv) 3.0 mM HU, (v) 3.5 mM HU and (vi) 4.0 mM HU. Histograms were generated using FlowJo^TM^ Sofware 10.5.3^35^.

We tested the effect of treating the roots with 2.0, 2.5, 3.0, 3.5 or 4.0 mM HU. The HU did not totally block DNA synthesis at any of the concentrations tested (Fig. 1), however it is not necessary to incubate the root tips at concentrations that block DNA synthesis for the whole period of incubation, as the degree of synchrony is not impaired if the cells escape the block at a certain HU concentration threshold and start synthesizing DNA in the presence of HU^20^. At 2.0 mM HU the effect is minor while at 2.5 mM HU there is some evidence of the HU arresting the cells at the G1 and early S phase. The coloured portion of the G2 peak in 3.5 mM histogram is misleading (Fig. 1v), the actual peak was slightly higher. There are therefore almost no differences in results for the 3.0, 3.5 and 4.0 mM HU concentrations. Treatment with 3.0 to 4.0 mM HU resulted in the highest S phase with the lowest G2 phase, indicating that these concentrations were the most effective at arresting the cells at the G1/S phase interface. We didn’t test concentrations of HU above 4.0 mM because at 4.0 mM some roots showed signs of stress and damage. Two additional genotypes responded to 3.0 and 4.0 mM HU treatments in a similar manner to the genotype shown in Fig. 1, with evidence of blocking at the G1/S interphase (Supplementary Fig. S1)

**Figure 2:**
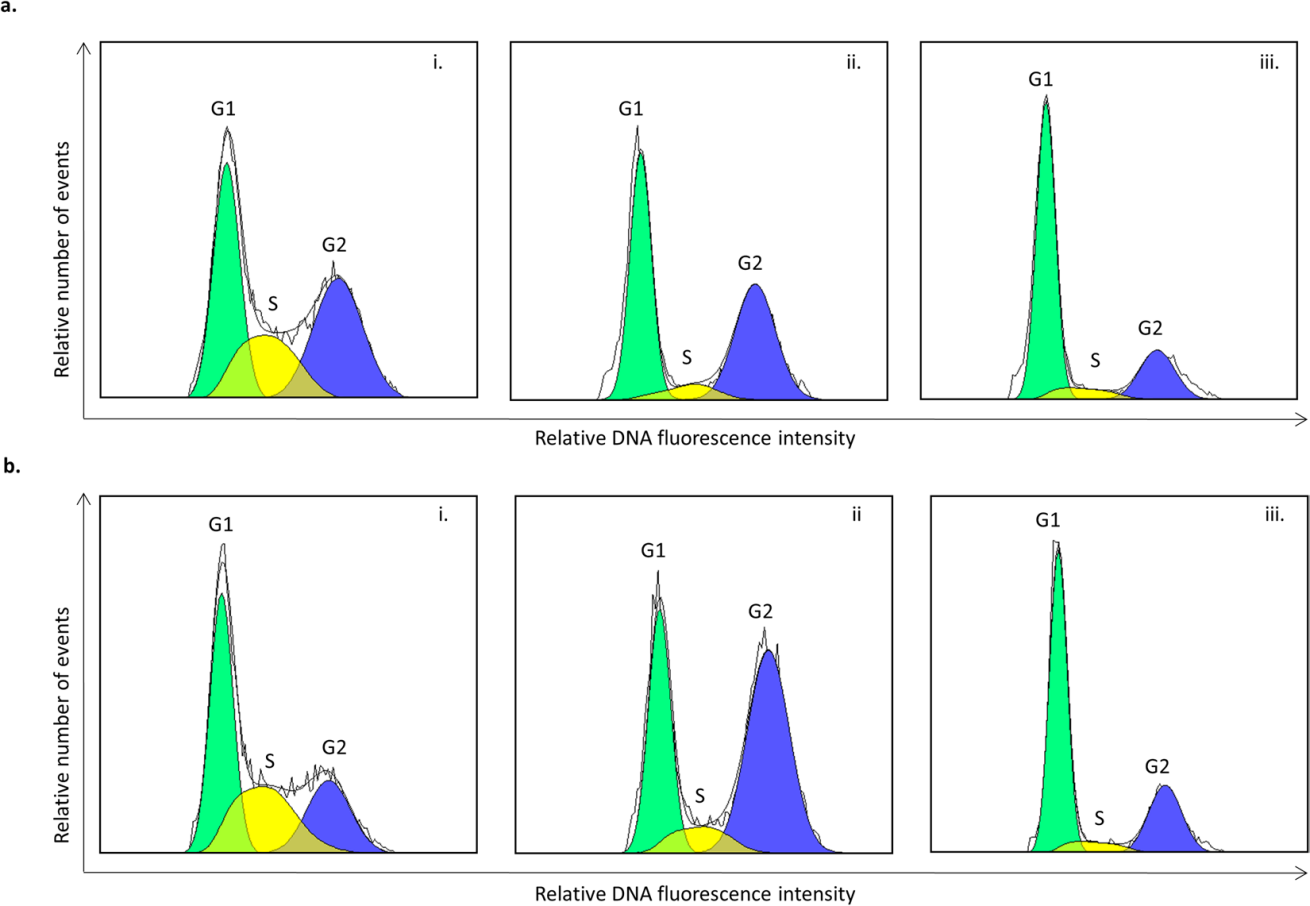
Cell cycle kinetics in root tip cells after the treatment with hydroxyurea (HU) at 2 concentrations. Sugarcane setts were treated in HU for 18 hours. (**a**) 3.0 mM HU, (**b**) 4.0 mM HU. (i) immediately after removal from the HU treatment, (ii) two hours and (iii) four hours after removal of the HU. Histograms show relative DNA content (linear scale) of nuclei in G1 (green), S (yellow) and G2 (blue) phases. Histograms were generated using FlowJo^TM^ Software version 10.5.3^35^.

Recovery time was initially estimated from a cell cycle analysis. After release from the HU block, cells traverse the S and G2 phases synchronously and enter mitosis. The time at which the G2 phase is the highest can therefore be used to estimate the time of highest mitotic activity. After two hours recovery both 3.0 mM and 4.0 mM HU resulted in an increased number of nuclei in G2 (Fig. 2aii,bii). After four hours most of the cells were in the G1 phase of the cell cycle (Fig. 2aiii,biii).

It is recommended that treatment with the mitotic spindle inhibitor should be started 0.5 hour to 1.5 hours before the peak of mitotic activity^19^. From Fig. 2, we estimated that the peak mitotic activity would be around 2 hours (when G2 phase peak was highest), so we tested one, two and three hours recovery followed by 3 hours in 2.5 µM or 5 µM APM using root tip squashes. The highest mitotic index was for two hours recovery followed by 5 µM APM (27%) and for one hour recovery followed by 2.5 µM APM (26%) (Supplementary Table S1). We estimated the mitotic rather than a metaphase index, because it was difficult to distinguish the different stages of mitosis with certainty. A metaphase index of at least 50% is recommended^21^, however the highest frequency of cells in mitosis we were able to achieve was not quite 30%. The parameters for the synchronisation treatment was set to 3.5 mM HU for 18 hours, one hour recovery and 2.5 uM APM for 2.5 to 3 hours. The concentration of HU was dropped to 3.5 mM because at 4.0 mM there were many roots with red bands, an indication of stress, which were unusable.

### Preparation of intact chromosome suspension

Three percent formaldehyde resulted in mostly well-fixed chromosomes, but with some evidence of poorly fixed chromosomes in some areas (Table 1 and Supplementary Fig. S2). At 4% there was evidence of chromosome clumping, and at 5% of intact cells (Table 1 and Supplementary Fig. S2).

**Table 1:** Quality of suspensions at a range of formaldehyde concentrations and 2 fixative incubation times, after a 2-step treatment with 3.5 mM HU for 18 hours, 1 hour recovery followed treatment with 2.5 µM APM for 3 hours.

Concentration formaldehyde (%)	Fixation incubation time (mins)	Result
2	20	poorly fixed nuclei and chromosomes
3	20	mostly well-fixed chromosomes
4	20	mostly well-fixed chromosomes, with some chromosome clumps
5	20	intact cells as well as well-fixed chromosomes
2	25	poorly fixed nuclei and chromosomes
3	25	mostly well-fixed chromosomes, with some clumps

Mechanical disruption of cells was by homogeniser at 18,000 rpm for 18 sec. The quality of the suspensions was assessed by dropping an aliquot onto a microscope slide and examining under a fluorescence microscope. Examples of the quality of suspensions are shown in Supplementary Fig. S2.

The concentration of the Triton-X100 in the fixative was compared at 0.1% and 0.05%, in addition, three speed settings on the homogeniser (14,000 rpm, 16,000 rpm and 18,000 rpm) were compared. At a concentration of 0.5% Triton-X100 in the fixation and the homogeniser speed set to 16,000 rpm the peaks in the flow karyotype was the clearest and there were mostly well-fixed chromosomes on the slides (Table 2 and Supplementary Fig. S3).

**Table 2:** Quality of flow karyotypes and chromosomes at 0.1% and 0.05% Triton-X100 in the fixative, and mechanical disruption by a homogeniser at three speed settings, after a 2-step treatment with 3.5 mM HU for 18 hours, 1 hour recovery followed by treatment with 2.5 µM AMP at 2.5 hours

% Triton-X100	Speed setting on homogeniser (rpm)	Quality flow karyotype	Quality chromosome morphology
0.1	14000	poorly defined peaks	mostly well-fixed chromosomes, some damaged chromosomes
0.1	16000	well defined peaks	mostly well-fixed chromosomes, some damaged chromosomes
0.1	18000	poorly defined peaks	mostly well-fixed chromosomes, some damaged chromosomes
0.05	14000	defined peaks	mostly well-fixed chromosomes
0.05	16000	clearly defined peaks	mostly well-fixed chromosomes
0.05	18000	defined peaks	mostly well-fixed chromosomes

Fixative parameters were 3.5% formaldehyde with 25 min incubation time. The quality of the suspensions was assessed by the quality of the flow karyotypes and the chromosomes flow sorted onto a microscope slide and examined under a fluorescence microscope. Examples of the quality of the flow karyotypes and chromosome morphology are shown in Supplementary Fig. S3.

### Identification of peaks containing chromosomes

**Figure 3:**
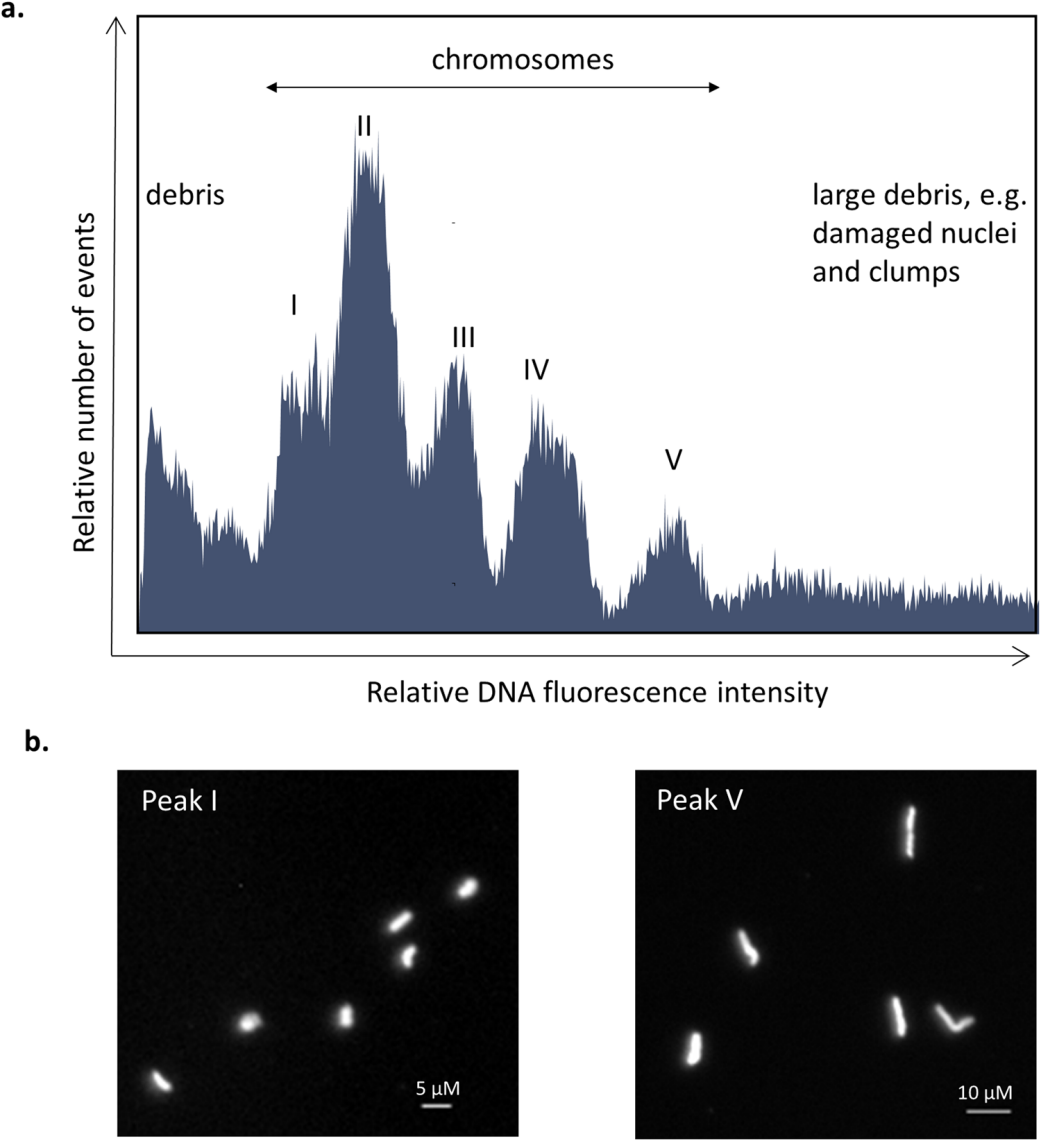
Histogram of relative DNA fluorescence of chromosomes (flow karyotypes) obtained by flow cytometric analysis of DAPI-stained chromosome suspensions of *S. officinarum*, Badila. (**a**) Flow karyotype, (**b**) images of chromosomes from peaks I and V flow sorted on microscope slides showing the difference in chromosomes size in the peaks with the smallest and largest chromosomes. Chromosomes were captured and digitized using a Carl Zeiss AxioImager M2 Fluorescence microscope with an Axiocam 506 Mono camera and Zen 2 Pro (blue edition) version 2.0.0.0 imaging software (https://www.zeiss.com/microscopy/int/products/microscope-software/zen.html).

To determine which peaks in the DAPI-Area (A) histogram represented chromosomes, 2,000 to 3,000 events were flow sorted onto microscope slides from a series of 10–12 sorting gates set on a histogram of relative fluorescence intensity obtained from the DAPI-stained chromosome suspension. Figure 3. shows a summary of results for the *S. officinarum* genotype Badila. Chromosomes were identified in a region containing five peaks. At lower fluorescence intensities debris was identified, while at higher fluorescence intensities damaged nuclei and clumped chromosomes were observed. Separate peaks containing debris, clumps and damaged nuclei in a flow karyotype from a root tip preparation is consistent with published results for wheat, although we were unable to identify individual peaks containing chromatids^22^.

### Chromosome histograms

**Figure 4:**
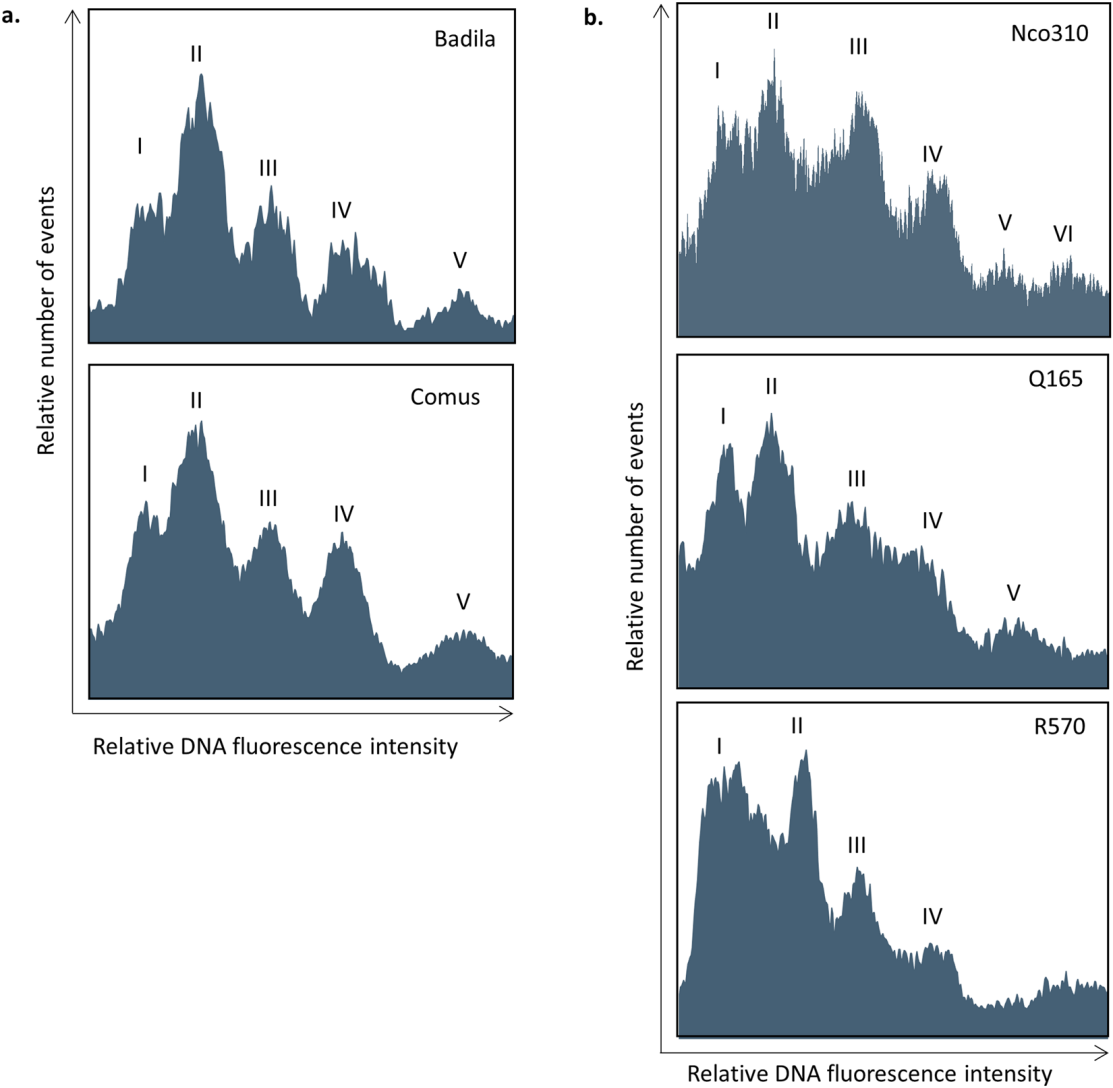
Histogram of relative DNA fluorescence of chromosomes (flow karyotypes) obtained by flow cytometric analysis of DAPI-stained chromosome suspensions. (**a**) Two S. *officinarum* genotypes, Comus and Badila, (**b**) three hybrid cultivars, Nco310, Q165 and R570.

Five main peaks, representing groups of chromosomes of similar DNA content or size, were identified in all five genotypes (Fig. 4). The peaks are more distinct in the two *S. officinarum* genotypes, Comus and Badila (Fig. 4a) than in the cultivars (Fig. 4b). Flow karyotypes were independently generated at least twice for all genotypes, except for Comus. For each genotype the flow karyotype profile was distinct and consistent. The two *S. officinarum* genotypes have similar profiles, with an additional peak V, peak II is the highest peak, and peak V is the lowest (Fig. 4a). For the three hybrid cultivars, the peaks are less defined and the profile for each is slightly different. Unlike the *S. officinarum* genotypes, peak I in the three hybrid cultivars is almost as high as peak II. Q165 has an additional peak V, like the *S. officinarum* genotypes, while we were unable to identify a peak V at all in R570 (Fig. 4b). A flow karyotype for Nco310 was generated twice, the first time a peak V was not distinct in the histogram (Supplementary Fig. S4), but there was a distinct cluster in a dot plot of 488-side scatter (SSC) vs DAPI-Area (A) (not shown). In the second flow karyotype generated, this peak was resolved into two separate peaks, peaks V and VI (Fig. 4b). In Nco310 Peak III is almost as high as peaks I and II, while in the other two hybrid cultivars, Q165 and R570, it is lower, similar to peak III in the *S. officinarum* genotypes. In Q165 peaks III and IV almost merge together completely, while they are more distinct in the other two hybrid cultivars.

### Assignment of chromosomes to peaks on flow karyotypes

For each genotype, except for R570, 40,000 to 50,000 chromosomes from each peak were flow sorted, purified and amplified. Yields and molecular weight of the amplified peaks were within the expected ranges, 25–35 µg per 50 µL reaction (Qiagen REPLI-g Advanced DNA single Cell Kit Handbook), except for the yields for the Badila peaks, which were lower than expected. Purified DNA from each peak were screened with 10 SSRs, one located on each of the 10 basic chromosomes aligned to Sorghum. Peak III from Q165 was split into two peaks, IIIa and IIIb; while peaks V and VI from Nco310 were flow sorted and analysed as a single peak. The SSR allelic bands were scored from the autoradiographs as present or absent for each of the peaks.

As sugarcane is an autopolyploid each SSR detected from seven to 14 alleles across the four genotypes. Each SSR detected from one allele (SMC1069 in Badila) to nine alleles (CIR60 in Q165). This variation in allele number detected is a combination of the ploidy level of the genotype (octoploid for *the S. officinarum* genotypes and greater for the hybrid cultivars), plus the polymorphism level and therefore dosage of each allele. CIR60 detected many single dose alleles on each homologous chromosome whereas SMC1069 has a low polymorphism level in *S. officinarum* genotypes, so detects only one allele as a multidose marker. When comparing alleles detected in the total genomic DNA for each genotype (Table 3) to the DNA from sorted chromosomes from each of the peaks it was apparent that all peaks contained at least one allelic copy of all SSR markers from the 10 basic chromosomes (Table 3). This indicated that each peak contained at least one homo(eo)log of each of the 10 chromosomes. It was also apparent that for the majority of the SSR markers different alleles were missing from each of the peaks. This was clearer in the *S. officinarum* genotypes due to the lower ploidy level and the simpler genome. For example, in Comus four alleles were detected for CIR60 in total genomic DNA, but in peak I allele five and 11 were missing but the other two alleles were present, in peak II allele two and six were missing, peak III allele 11 was missing, peak IV alleles five and 11 were missing and peak V alleles two and five were missing. In Q165 the same SSR identified nines alleles and for each peak different combinations of alleles were present/absent. The number of alleles missing from each peak ranged from one to seven (Table 3).

**Table 3:** Analysis of number of alleles present/absent from each SSR markers identified in each of the five peaks for the two *S. officinarium* genotypes examined, Badila and Comus.

Genotype	Sorghum Chromosome	Genome		Peak											
				I		II		III		IIIb		IV		V	
		# AP	AP	AP	# AA	AP	# AA	AP	# AA	AP	# AA	AP	# AA	AP	# AA
Badilla	1	5	3,4,5,6,7	5,6,7	2	3,4,5,7	1	4,5,6	2			3,4,5,7	1	3,5,6,7	1
	2	3	1,5,6,	1,6	1	5,6	1	1,5,6	0			5,6	1	1,5,6	0
	3	7	1,2,5,6,10,13,14	2,6,10	4	2,10,13	4	5,6,10,14	3			1,2,5,6,10	2	1,2,5,6,10	2
	4	5	1,2,6,8,10	2,8,10	2		—	1,6,8,10	1			1,2,6,8,10	0	6,8,10	2
	5	5	2,3,4,5,6,	2,3,4,5,6	0	2,3,4,5,6	0	2,3,4,5,6	0			2,3,4,5,6	0	2,5,6	2
	6	6	3,4,5,6,8,10	3,6,8,10	2	4,5,6,8,10	1	6,8,10	3			4,5,6,8,10	1	3,4,5,6,8,10	0
	7	1	4	4	0	4	0	4	0			4	0	4	0
	8	4	2,3,4,5	2,3,4,5	0	2,5	2	2,5	2			2,3,4,5	0	2,3,4,5	0
	9	3	1,2,4	2,4	1	1,2,4	0	1,4	1			1,2,4	0	1,4	1
	10	4	1,4,5,8	1,5,8	1	4,5,8	1	5,8	2			1,5,8	1	4,5,8	1
Comus	1	4	2,4,5,6	2,5,6	1	2,5	2	2,5,6	1			2,4,5,6	0	2,5,6	1
	2	4	2,5,6,11	2,6	2	5,11	2	2,5,6	1			2,6	2	6,11	2
	3	6	3,5,6,11,13,14	5,11,14	3	3,5,6,11,13,14	0	3,5,6,11,14	1			5,6,11	3	3,5,11,14	2
	4	5	1,2,3,5,6	2,3	3	5,6	3	1,3,5,6	1			1,2,3,6	1	3,6	3
	5	4	1,2,3,5	2,3,5	1	1,2,3,5	0	1,2,3,5	0			1,2,3,5	0	1,2,3,5	0
	6	6	1,2,3,6,8,10	1,6,8,10	2	2,3,6,8,10	1	2,3,6,8,10	1			2,3,6,8,10	1	1,2,3,6,8,10	0
	7	1	4	4	0	4	0	4	0			4	0	4	0
	8	4	1,4,5,9	5	3	1,4,5,9	0	5	3			5	3	1,5	2
	9	2	1,4	1,4	0	1,4	0	1,4	0			1,4	0	1,4	0
	10	3	3,6,10	6,10	1	3,6	1	3,6,10	0			3,10	1	6,10	1
Nco310	1	5	2,4,5,7,9	2,4,5,7,9	0	2,4,5,7,9	0	2,5,7,9	1			2,7,9	2	2,4,5,7	1
	2	9	1,2,3,4,5,7,11,12,13	1,2,3,7,11,12,13	2	2,3,4,5,11,12,13	2	2,4,7,11,12,13	3			1,2,4,7,11,12,13	2	2,3,4,11,12	4
	3	8	2,3,4,7,8,9,10,13	2,3,7,9,10	3	2,3,8,9,10,13	2	2,3,4,7,10	3			2,3,8,9,10	3	2,3,4,7,8,9,10,13	0
	4	6	1,4,5,11,12,13	1,4,5,11,12	1	11,13	4	1,4,5,12,13	1			1,5,12,13	2	5,11,12,13	2
	5	6	1,2,3,4,5,6	2,3,4,5,6	1	1,2,3,4,5,6	0	1,2,3,4,5,6	0			1,2,3,4,5,6	0	1,2,3,4,5,6	0
	6	7	3,5,6,7,8,9,10	3,8,10	4	5,6,8,10	3	8,10	5			7,8,9	4	3,5,8,10	3
	7	3	1,4,5	1,4,5	0	1,4	1	4,5	1			4	2	1,4	1
	8	5	2,4,5,6,8	2,4,5,8	1	2,4,5,8	1	2,4,5,6,8	0			2,4,5,6,8	0	2,4,5,6,8	0
	9	4	3,3a,4,5	4	3	3,3a,4	1	3,3a,5	1			3,4,5	1	3,4,5	1
	10	7	2,4,5,6,9,10,11	2,6,9,11	3	4,5,9,11	3	4,5,10,11	3			2,4,5,6,9,10,11	0	2,4,5,6,9,10,11	0
Q165	1	5	1,2,5,7,8	1,2,5,7	1	1,2,5,7,8	0	1,2,5,7	1	1,2,5,7	1	2,5,7,8	1	1,2,5,7	1
	2	9	4,5,6,7,8,9,10,12,13	4,6,9,12,13	4	4,5,6,7,9,12,13	2	4,6,7,8,9,12,13	2	7,8,10	6	4,6,7,12,13	4	6,9	7
	3	7	2,4,6,10,11,12,14	2,4,6,11,12	2	4,6,10,11,12	2	4,6,11,14	3	2,4,6,11,12,14	1	2,10,11,12,14	2	2,4,6,10,11,12	1
	4	5	1,7,9,11,14	1,7,9,14	1	7,11,14	2	1,9,14	2	1,7,9,11,14	0	7,9,11,14	1	9,11	3
	5	4	1,3,4,7	1,3,4,7	0	1,3,4,7	0	1,3,4,7	0	1,3,4,7	0	1,3,4,7	0	1,3,4,7	0
	6	7	2,3,5,6,8,10,11	2,3,5,6,8,10,11	0	5,6,8,10	3	2,3,5,6,8,10,11	0	2,3,5,6,8,10,11	0	2,3,5,6,8,10,11	0	2,3,5,6,8,10	1
	7	6	2,3,4,5,6,7	2,3,4,5,6,7	0	4	5	2,3,4,5,6,7	0	4,5,6	3	2,3,4,5,6,7	0	2,3,4,7	2
	8	7	2,3,4,5,6,7,9	2,3,4,5,7	2	2,3,4,5,7,9	1	2,3,4,5,7,9	1	2,3,4,5,7	2	2,3,4,5,7	2	2,3,4,5,6,7	1
	9	6	1,2,3,4,4a,5	1,2,4,5	2	1,2,3,4,4a	1	1,2,4,5	2	1,2,3,4,5	1	1,4,5	3	1,2,3,4,5	1
	10	7	4,5,6,7,8,9,10	6,7,8,9	3	5,7,9,10	3	5,7,9,10	3	4,5,7,9,10	2	4,5,7,10	3	5,6,7,9,10	2

The peak numbers refer to the flow karyotype as shown in Fig. 4 and represent groups of chromosomes of similar DNA content. # AP = Number of Alleles Present, AP = Alleles Present, # AA = Number of Alleles Absent.

In many cases there is no missing allele and that is because the alleles are present in more than one copy and are present on more than one homologous chromosome so appear in more than one peak e.g. Q165 chromosome 3 and chromosome 5. If enough polymorphic SSRs were screened the complement of chromosomes present in each peak could be resolved, but a very large number of SSRs would have to be screened that contained polymorphic single or low dose bands. In only a few cases was there only one allele present in a peak. For example for Nco310, CIR74, only allele four was present in peak I but, as only a total of four alleles were identified for this SSR, and we know that Nco310 has over eight copies of chromosome nine, these alleles are present on at least two chromosomes. It can be seen from Table 3 that the allelic patterns in Badila and Comus are less complex as they are *S. officinarum* genotypes and are octoploid, unlike the cultivars which have inherited *S. spontaneum* chromosomes and are at a higher ploidy level. Unfortunately, as the polymorphism level is also lower, with always less than eight alleles identified in most cases, the alleles are present on more than one copy of a homologous chromosome.

## Discussion

Many economically important crop species have large and complex genomes^23^ which challenges our ability to understand and manipulate them. One strategy that has been successfully used in wheat is to simplify the genome by physically breaking it down into single chromosomes or groups of chromosomes^17^. The most successful method developed uses HU and AMP or oryzalin to accumulate dividing cells at metaphase and mechanical homogenization to isolate chromosomes^19^. The resulting suspension of intact chromosomes can be classified according to relative DNA content by flow cytometry to create histograms or flow karyotypes. Chromosomes or groups of chromosomes of a similar DNA content group together in peaks, which can be individually flow sorted and examined. Here we modified the method of Vrána *et al*.^19^ to break down the highly polyploid and complex genome of the economically important crop, sugarcane.

The conditions described by Vrána *et al*.^19^ for sugarcane resulted in a very low mitotic index in our hands. For each species both the HU concentration and the recovery time (time between the release of the HU block and treatment in the mitotic spindle inhibitor) needs to be optimised. The concentration of the HU and the recovery time were estimated from cell cycle analyses (Figs. 1 and 2). Root tip squashes were used to determine the optimal APM concentration and incubation time. The final parameters used for cell cycle synchronisation were: 18 hours in 3.5 mM HU, 1 hour recovery, 2.5 hours in 2.5 µM APM. The resulting HU concentration was nearly twice that recommended and the recovery time much shorter^19^. Similarly short recovery times using the same method with HU and APM have been reported for an oat-maize line^13^. However, although the resulting concentration of chromosomes in suspension was lower than that recommended^19^, we were able to produce histograms and flow sort at least 3 × 10^5^ chromosomes from 100 root tips. Independently generated histograms from the same genotype resulted in histograms with similar profiles (Supplementary Fig. S4).

Histograms from flow sorted chromosomes show the number of chromosomes vs the relative area of the chromosomes, which is a proxy for the size of the chromosome. The chromosome histograms, or flow karyotype, for the two *S. officinarum* genotypes are similar, suggesting that there is a similar grouping of chromosomes according to size (Fig. 4a). These results are consistent with all *S. officinarum* genotypes examined having a chromosome complement of 2n = 80^10^. The largest chromosomes fall into the smallest peak (Fig. 4a peak V), that is, the smallest number of chromosomes are the largest in size. There are three peaks about the same height, representing three groups of chromosomes that have about the same number of chromosomes (Fig. 4a peaks I, III and IV). These are a group of the smallest chromosomes (peak I), and the 3^rd^ and 4^th^ largest chromosomes (peaks III and IV). The highest peak, representing the largest group, are the 2^nd^ smallest chromosomes (peak II).

There are two main differences between the flow karyotypes for the hybrid cultivars and *S. officinarum* genotypes. First, the peaks in all the hybrid cultivar histograms are less discrete than those from *S. officinarum*. These results can be explained by the hybrid genome content of modern genotypes. Modern cultivars are derived from initial hybrids between the two *Saccharum* species followed by crossing either to *S. officinarum* or cultivars to recover the agronomically acceptable phenotype. The two contributing species have different basic chromosomes numbers and, although there is limited information on chromosome pairing in sugarcane, genetic linkage maps have identified translocations that involves six of the *S. officinarum* chromosomes^13,24,25^ and accounts for the reduction in the basic chromosome number from x = 10 to x = 8. These translocations were confirmed in the recently published monoploid genome sequence of sugarcane cultivar R570^13^ and the haploid genome sequence of a *S. spontaneum*^26^ and this would account for the larger variation in chromosomes sizes in genotypes and the resultant increased merging of the peaks in the flow karyotype. Unlike *S. officinarum, S. spontaneum* genotypes have a wide range of complement numbers, from 2n = 40 to 128^14,15^. Modern hybrid cultivars have complement numbers from 2n = 100 to 120 and varying proportions of *S. spontaneum* (10–23%) and recombinant chromosomes (8–13%)^14^. The differences between the *S. officinarum* and hybrid cultivar histograms are due therefore to the presence of *S. spontaneum* and *S. officinarum-S. spontaneum* recombinant chromosomes. Interestingly there was more variation in flow karyotypes between the hybrid cultivars than is seen in other species, such as wheat. Differences between the hybrid cultivar histograms are probably due to a combination of two factors which interact in three ways: first, different *S. spontaneum* genotypes used in the initial crosses; second, the retention of different *S. spontaneum* chromosomes in the subsequent backcrosses, and third, genomic remodelling, including variation in recombinant chromosomes^27^. Unfortunately, we were not able to generate histograms for *S. spontaneum* because we were unable to obtain enough rapidly growing roots. The morphology of *Saccharum spontaneum* is highly polymorphic, ranging from small grass-like plants without stalks to plants over five meters high with long thin stalks^28^. The thin *S. spontaneum* stalks grown under the same conditions as those we used for *S. officiniarum* and the hybrid cultivars resulted in slower growing roots from which we were unable to isolate chromosomes to generate a flow karyotype.

*Saccharum officinarum* and modern hybrid cultivars have a basic chromosome number of 10 ^29,30^. Given that there are four-six peaks in the flow karyotypes, at least one peak, and probably more than one peak, in each flow karyotype must represent more than one homo(eo)log of a given chromosome. SSR markers were used to examine the chromosomal component of each peak in all genotypes except R570. All peaks contained at least one homo(eo)log of each of the 10 chromosomes, a single peak therefore does not contain all or even most homo(eo)logs of a particular chromosome. Wheat is the closest relative to sugarcane with the best studied flow karyotype. The flow karyotype for common wheat has four peaks, and the three genomes fall very roughly into the three main peaks. Peak I is composed of four chromosomes from the D genome, peak II of chromosomes from A and D genomes, peak III of chromosomes from the A and B genomes, while peak IV is the 3B chromosome^31^. Our results suggest that there may be a similar situation in sugarcane, complicated by its double genome structure. The SSR markers show that although all peaks contain all chromosomes there were alleles from each marker missing from each peak. This suggests a structure to the *Saccharum* genome not previously identified, composed of five groups based on size, with each group containing one or more homo(eo)logs of a particular chromosome. This indicates that it is possible each peak represents an ancestral sub genome or more likely sub-genomes that have gone through a whole genome duplication. The delineation between these sub-genomes has been obscured by the autopolyploid pairing of *Saccharum*. The indication of sub-genomes from the karyotype analysis does start to explain the combination of auto/allo ploidy that has been documented in *S. officinarum* genetic maps^32,33^, where preferential pairing was detected.

We have modified the protocol of Vrána *et al*.^19^, to generate a high mitotic index for sugarcane and produce flow karyotypes for both *S. officinarum* genotypes and a number of sugarcane hybrid cultivars. This is the first time a flow karyotype has been generated for sugarcane. *S. officinarum* generated sharper and cleaner peaks than the varieties. This is expected as *S. officinarum* is a balanced octoploid so fewer chromosome size variations are likely. The karyotypes for the hybrid cultivars contained less defined peaks which is probably due to the hybrid cultivars having chromosomes from both *S. officinarum* and *S. spontaneum* plus recombinant chromosomes. Interestingly, there was more variation in the karyotypes between the varieties than is seen in other species which could be explained by variation in *S. spontaneum* chromosomes, recombinant chromosomes between the two species and the fact that all varieties are aneuploid. SSR analysis indicates that the peaks contain at least one copy of each of the 10 chromosomes and may represent ancestral *Saccharum* subgenomes or genomes. The ability to flow sort *Saccharum* chromosomes provides the possibility of isolating and analysing chromosomes of interest, for example, chromosomes carrying genes associated with disease or pest resistance, or examining synteny between and the structure of homo(eo)logous chromosomes and genes.

## M&M (max 1500 words, this is 1646)

### Plant material

Two *S. officinarum*, Comus and Badila, and three hybrid cultivars, R570, Q165 and Nco310, were examined. Q165 is an Australian commercial variety bred by Sugar Research Australia, Nco310 was bred in Coimbatore, India, and was the most important cultivar worldwide in the 1950s and 1960s^34^, while R570 was bred by CERF (now eRcane) in Reunion and has the best-characterised sugarcane genome to date^30^. Badila was one of the original *S. officinarum* genotypes grown for commercial sugar and used to generate the first hybrids.

All five genotypes were grown in the field at the CSIRO Gatton field station (Queensland) or in a glasshouse at CSIRO (St Lucia, Queensland) with a natural photoperiod, 12 hours 30 °C, 12 hours 24 °C and humidity >55%/70% (day/night relative humidity).

### Cell cycle synchronisation

Sugarcane is routinely propagated by setts, single bud sections of sugarcane stalks with root primordia. Sett germination and optimisation of the two-step synchronization procedure were carried out in a Conviron A100 growth chamber with a CMP6010 control system set at 32 °C in the dark. All solutions, including water for washes, were maintained at 32 °C +/− 0.5 °C. The setts were grown in moist peatmoss and perlite at a ratio of 0.05 L: 1 L for 71–77 hours until the roots were 1–3 cm long. Before treatment, they were then removed and washed in water. Setts were treated in HU, water and AMP with a maximum of one sett per 0.2 L of solution and well aerated with air pumps and air stones. The HU and APM were dissolved in deionised water, rather than Hoagland’s nutrient solution which is usually used^19^. Sugarcane setts grow equally well in water as in Hoagland’s nutrient solution (data not shown).

Initial estimates for the optimal HU concentration and recovery times were determined by cell cycle analysis using flow cytometry. Nuclei were isolated for cell cycle analysis as described by Vrána *et al*.^19^. The analysis was performed on a BD BioSciences LSR II equipped with a 355-nm true-UV laser with 100-mW output power for DAPI excitation and 488-nm laser for scattered light detection. DAPI fluorescence from the stained nuclei was collected through a 448/59 band-pass filter. For the cell cycle analysis, gates were first set on a dot plot of forward scatter (FSC)-Area (A) vs side scatter (SSC)-A to exclude debris and then a tight gate set on a dot plot of DAPI-Height (H) vs DAPI-Width (W) to exclude doublets. Relative fluorescence intensities, which corresponded to relative DNA content of gated populations, were acquired on histograms of DAPI-A. 10,000 nuclei were acquired for each sample. The resulting fcs files were analysed using FlowJo software version 10.5.3^35^ with the Dean-Jett fox model and the G2 peak constrained between 1.8 and 1.9 of the G1 peak.

Estimates of the optimal HU concentration were determined by treating setts in 2.0, 2.5, 3.0, 3.5 or 4.0 mM HU for 18 hours, and then transferring them to water at 32 °C. The optimal hydroxyurea concentration was determined as the concentration that resulted in the largest S phase component at zero hours recovery without compromising root morphology. The HU concentration was tested for three genotypes.

Once it had been determined that the three genotypes responded in a similar way to the HU, a time series after HU treatment was undertaken to estimate the optimal recovery time. Recovery time is the length of time between samples being released from the HU and being treated in the APM. It is recommended that treatment with the mitotic spindle inhibitor (APM) should be started 30 to 90 minutes before the peak of mitotic activity^19^. The time of peak mitotic activity was estimated from the cell cycle histograms. Setts were treated in two concentrations of HU for 18 hours, rinsed well in water at 32 °C and then transferred to aerated water at 32 °C. Samples were taken at time intervals, straight after being removed from the HU, 2 hours recovery and 4 hours recovery. Root tips were prepared immediately after removal from the water for cell cycle flow analysis.

To assess the degree of metaphase synchrony after treatment root tips squashes were prepared and imaged with a Carl Zeiss AxioImager M2 Fluorescence microscope equipped with filter set appropriate for DAPI. Images were captured and digitized using an Axiocam 506 Mono camera and Zen 2 Pro (blue edition) version 2.0.0.0 imaging software (https://www.zeiss.com/microscopy/int/products/microscope-software/zen.html). Setts were treated with 4.0 mM HU, with 1, 2 or 3 hours recovery followed by 3 hours in 2.5 µM or 5 µM APM. Squashes were prepared according to Vrána *et al*.^19^ with the following modifications. Instead of macerating the root tips in 45% acetic acid, root tips were digested in an enzyme mix of 2% cellulase, 2% pectinase in a digestion buffer (10 mM trisodium citrate-dihydrate, 10 mM citric acid-monohydrate, 75 mM KCl) for 15 minutes. After removal from the enzyme mix they were further stained in an 0.5% acetocarmine solution and squashed between a microscope slide and an 18 mm × 18 mm coverslip.

### Preparation of intact chromosome suspension

At the recommended formaldehyde fixation and tissue homogenisation parameters for sugarcane^19^, the morphology of a large proportion of the chromosomes was poor. We therefore modified the method described by Vrána *et al*.^19^. We tested increased concentrations of formaldehyde in 1% steps, half the concentration of the Triton X-100 in the fixative to 0.05%, increased fixative incubation times in 5 minute intervals, as well as reducing the speed of the homogeniser unit (Kinematica Polytron PT 1300 D) at approximately 2,000 rpm intervals. The chromosome suspension was stained with DAPI to a final concentration of 2 µg/mL. The quality of the chromosomes was assessed by dropping the suspension onto slides or by flow cytometry. Flow cytometry samples were filtered through a 25 µM nylon mesh and kept at 4 °C until the next day and then filtered a second time through a 10 µM nylon mesh just before it was run on the flow cytometer. Fixation conditions and homogenization times were assessed by quality of the histograms and of the chromosomes flow sorted or dropped onto slides. Slides were examined with a Carl Zeiss AxioImager M2 Fluorescence microscope equipped with filter set appropriate for DAPI and the images captured and digitized using an Axiocam 506 Mono camera and Zen 2 Pro (blue edition) version 2.0.0.0 imaging software (https://www.zeiss.com/microscopy/int/products/microscope-software/zen.html). The samples were checked for chromosome clumping and for damaged/poorly fixed nuclei and chromosomes. Chromosome clumps and intact cells indicate over fixing^19^.

### Flow analysis of chromosome suspension and identification of peaks containing chromosomes

Chromosome flow cytometric analysis and sorting was performed on a Beckman Coulter MoFlo AstriosEQ cell sorter equipped with a 355-nm true-UV laser with 100-mW output power for DAPI excitation and 488-nm laser for scattered light detection. IsoFLOW (Beckman Coulter) was used as sheath fluid. DAPI fluorescence from the stained isolated chromosomes was collected through a 448/59 band-pass filter. Gates were first set on a dot plot of DAPI-A vs FSC-A to exclude debris and then a tight gate set on a dot plot of DAPI-A vs DAPI-W to exclude doublets. Relative fluorescence intensities were acquired on a DAPI-A histogram.

To determine which peaks in the DAPI-A histogram represented chromosomes, rather than debris or damaged nuclei, a series of sorting gates were set on histograms of DAPI-A. For each gate 2,000–3,000 events were flow sorted onto slides and examined under the Carl Zeiss AxioImager M2 Fluorescence microscope.

### Assignment of chromosomes peaks on flow karyotypes using Simple Sequence Repeat (SSR) markers

To obtain DNA from chromosomes in each peak, approximately 40,000 to 50,000 chromosomes were flow sorted into a tube sorted at a rate of 50–200 events per second. Chromosomal DNA was purified according to Vrána *et al*.^19^, with the following modifications; 120 ng/µL of proteinase K (20 mg/mL Qiagen) was used at the first proteinase K digestion step, a Millipore Microcon DNA Fast Flow Device (Millipore) was used to remove the proteinase K, all centrifugations were done at 4 °C and the centrifuge speeds and times were performed according to Giorgi *et al*.^36^. After the final wash, the device was centrifuged at 500 × *g* 4 °C, for 5 minutes at a time, until the final volume on the column was less than 20 µL. DNA was quantified using the Qubit dsDNA HS Assay kit (ThermoFisher Scientific). 15 µL of the DNA from the purified chromosomes was amplified with the Qiagen REPLI-g Single Cell Kit, using Qiagen’s supplementary protocol ‘Whole genome amplification from genomic DNA using the REPLI-g Single Cell Kit with increase sample volumes’. Incubations were carried out for 16 hours. DNA was quantified with the Qubit dsDNA BR Assay kit (ThermoFisher Scientific) and visualized on an agarose gel.

### Identification of chromosomes in flow sorted fractions

SSR primers were obtained from the International Sugarcane Microsatellite Consortium collection^37^. The primers used are shown in Table 4.

**Table 4:** List of sugarcane SSR primers used to assign chromosomes to peaks in the flow karyotypes shown in Fig. 4.

Name	Sorghum chromosome	Forward primer	Reverse primer	Repeat Unit	Annealing Tm	EMBL accession number
mSSCIR19	1	GGT TCC AAA ATA CAC AAA	CAA TCT TAT CTA CGC ACT T	(GA)_23_	52	AJ293491
mSSCIR60	2	GGC TGC TGG CTG GGT TG	CAT CAT TCC GCC TGT CAT TG	(GT)_18_(GA)_9_	54	AJ401337
mSSCIR35	3	CTC ACT CAA GAA GCC ACA T	CCA TCA GCC AGC AGT CTC T	(GA)_11_N_80_(GA)_19_	54	AJ401312
mSSCIR77	4	CAA AAG CTG AAA TGG TCT C	TTG CCA CGA AAG ATA AAA C	(GT)_34_	46	AJ293563
mSSCIR28	5	CCG CAT CTC TTT GTT TTG	GGT GGT GAT GAG TCG TGA	(GA)_34_	54	AJ293499
mSSCIR46	6	ATG CTC CGC TTC TCA CTC	AAG GGG AAA ATG AAA ACC	(GT)_26_	54	AJ401323
SMC1069	7	TCA GGC GTT CAC AAG GCT T	GCT GCA CCT TCC CGT ATG A	(CA)_13_	54	
mSSCIR24	8	AGA TGA ACC CAA AAA CTT A	TTA CTC CGC CTC TTT ACT	(GA)_20_	54	AJ293495
mSSCIR74	9	GCG CAA GCC ACA CTG AGA	ACG CAA CGC AAA ACA ACG	(GGC)_9_	54	AJ401351
mSSCIR55	10	ATA TGT AGG AGT AGG ACC AA	CAA CAG GTT TCA GTA TAT TT	(GT)_30_	54	AJ401332

10 SSRs were amplified, one located on each of the ten basic chromosomes aligned to Sorghum. The name of the SSR primer, the chromosome it is located on, as aligned to Sorghum, the sequence, repeat unit amplified, annealing temperature used and EMBL accession number, where available, are shown.

SSR markers were generated using methods described in Aitken, Jackson & McIntyre (2005)^38^. PCR reactions were carried out in a final volume of 15 µL with 0.5 ng/µL of template, 3 mM MgCl_2_, 0.2 mM each of ATP, dGTP, dTTP, 0.02 mM dCTP, 0.67 mM each primer and 1.5 U of Taq. The SSR products were labelled during the PCR reaction with 0.06 uL of α-[33 P] dCTP (3,000 ci/mmol). Cycling conditions used were 94 °C for 1 minute, 35 cycles of 94 °C for 30 seconds, 54 °C for 1 minute, 72 °C for 30 second, followed by a final extension of 72 °C for 5 minutes. PCR products were denatured at 95 °C for 5 minutes and run on a 5% polyacrylamide denaturing gel at 90 W for 2 hours. The gels were then dried using a gel dryer for 25 minutes at 80 °C and exposed to Fuji RX-N X-ray film for 3–4 days.

Whole genomic DNA was extracted for each *S. officinarum* and hybrid cultivar using a standard CTAB method^39^, replacing the octanol with isoamyl alcohol. Whole genomic DNA and the five peaks from the four hybrid cultivars, Badila, Comus, Nco310 and Q165, were screened with the 10 SSRs, which had previously been assigned to the ten Sorghum chromosomes. The allelic pattern for each peak was compared against that of the whole genome sample.

## Supplementary information

Supplementary information
