# Supplementary material for: Flow cytometric characterisation of the complex polyploid genome of Saccharum officinarum and modern sugarcane cultivars
Source: Sci Rep. 2019 Dec 18;9:19362. doi: 10.1038/s41598-019-55652-3 (PMC6920420; doi:10.1038/s41598-019-55652-3)
Supplement: Supplementary file 1 — Supplementary information [file 41598_2019_55652_MOESM1_ESM.pdf]

Supplementary Table S1. Estimate of degree of synchrony. The mitotic index was estimated from squash preparations of sugarcane root tips after a 2-step treatment with 4.0mM hydroxyurea for 18 hours, recovery for 1, 2 or 3 hours, followed treatment with 2.5 µM amiprophos methyl for 3 hours.

Hours after hydroxyurea removal (recovery time)	Concentration AMP (µM)	Number of images examined	Number of cells examined	Average mitotic index
1	2.5	16	1504	26%
1	5	16	1917	23%
2	2.5	42	4274	20%
2	2.5	13	1408	22%
2	5	16	1636	27%
3	2.5	37	3070	23%

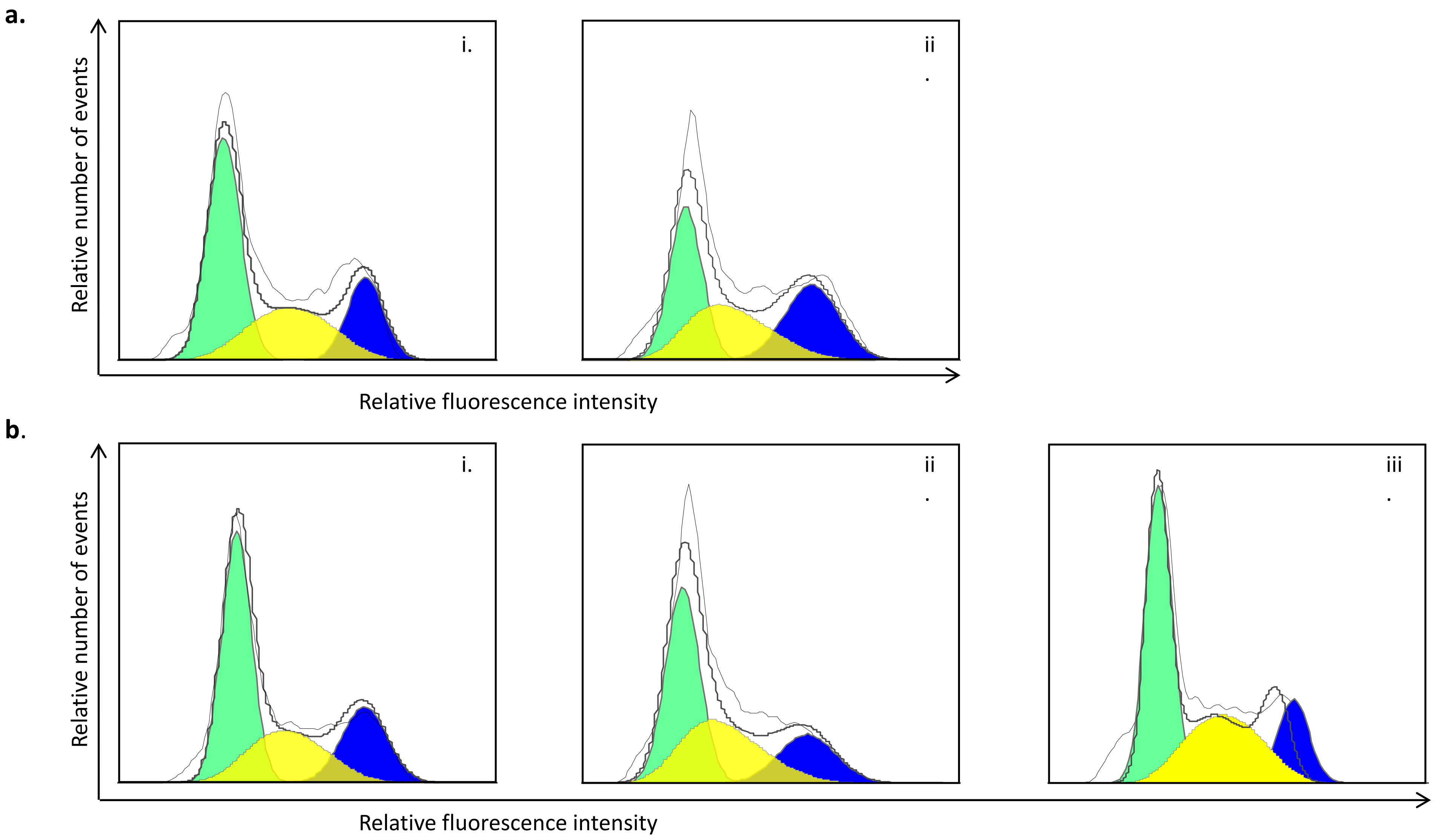

Figure S1. **The effect of hydroxyurea (HU) at 2 concentrations on cell cycle kinetics in root tip cells of 3 genotypes.** The first genotype tested (i) is shown for comparison. Setts were treated in HU for 18 hours, root tips excised and nuclei were isolated for flow cytometric analysis of DNA content immediately after removal from the HU treatment. Histograms show the relative DNA content (linear scale) of nuclei in G1 (green), S (yellow) and G2 (blue) cell cycle phases. a. = 3.0 mM HU, b. = 4.0 mM HU. i. = cultivar 1, ii. = cultivar 2, iii. = cultivar 3.

A. B. C.

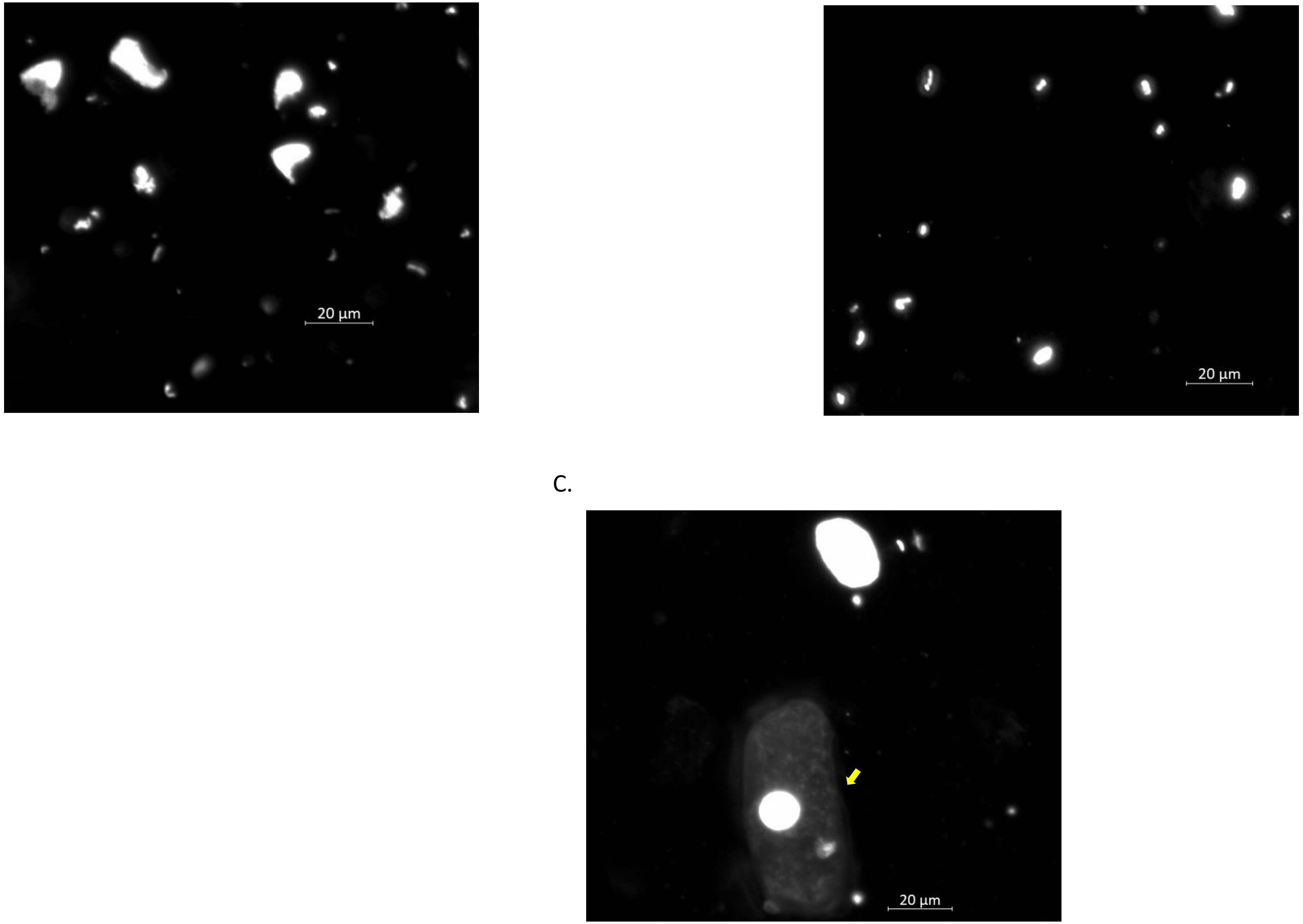

Figure S2. Examples of the quality of suspensions in Table 1. A. = Poorly fixed nuclei and chromosomes B.= Mostly well-fixed chromosomes C. = Intact cells as well as well-fixed chromosomes. The yellow arrow indicates an intact cell.

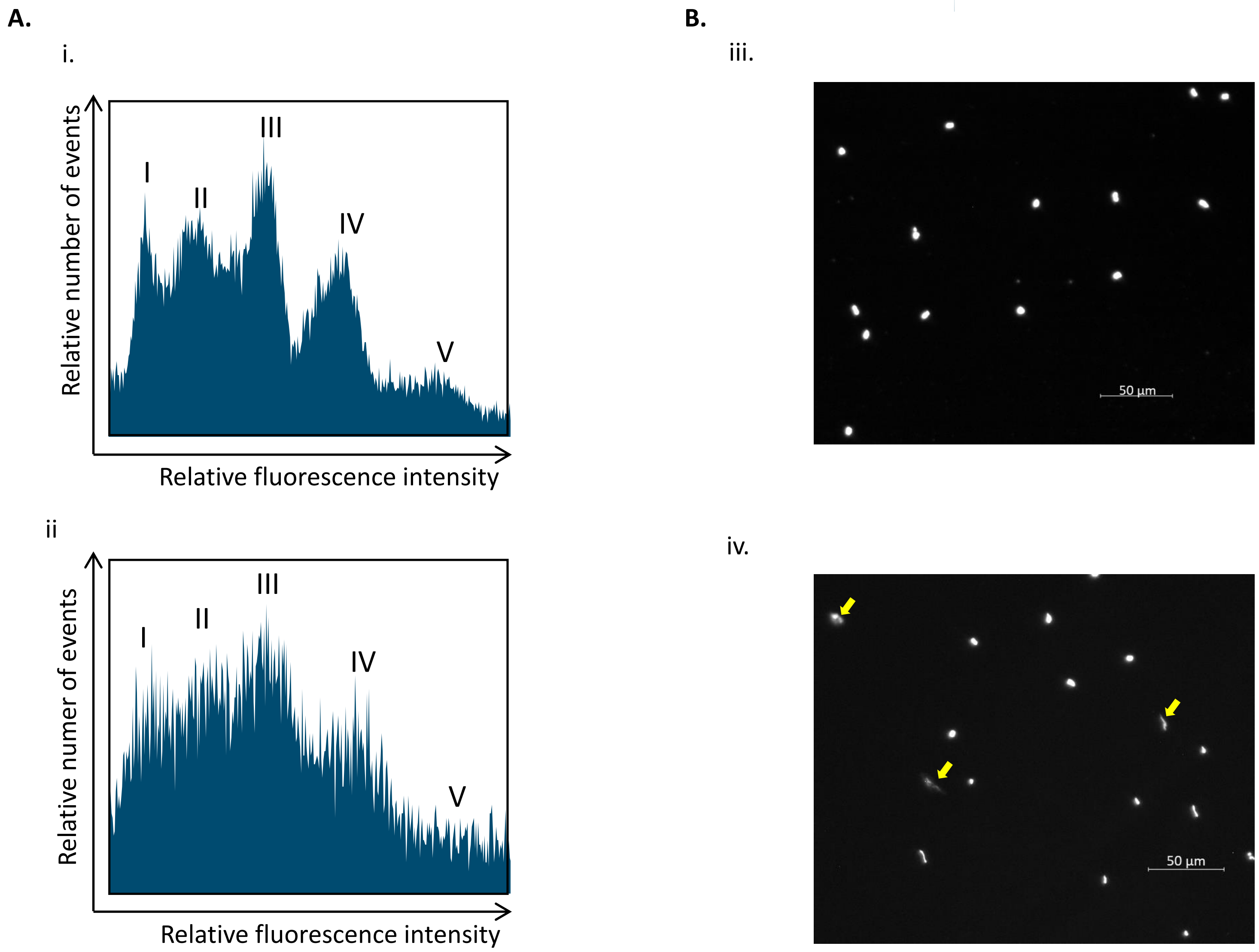

Figure S3. Examples of flow karyotypes and chromosome morphology in Table 2. a = flow karyotype, b= chromosome morphology. I = well defined peaks, ii = poorly defined peaks, iii = mostly well fixed chromosomes, iv = mostly well fixed, some damaged chromosomes. the yellow arrows indicate poorly fixed and damaged chromosomes.

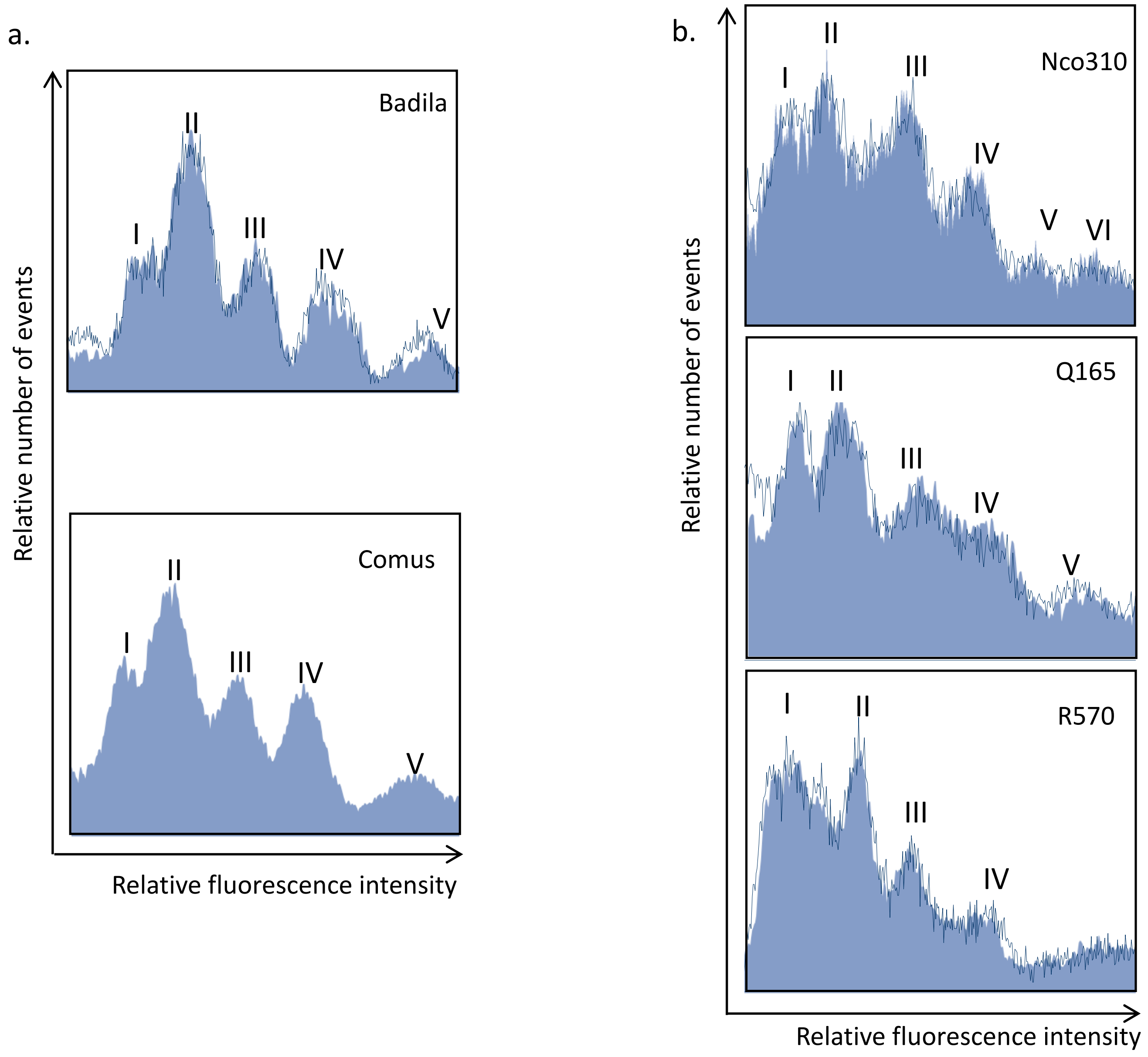

Figure S4. Duplicate histograms of relativeDNA fluorescence (flow karyotypes) obtained by flow cytometric analysis of DAPI-stained chromosome suspensions. a. = the *two S. officinarum* genotypes, Comus and Badila, b. = the three hybrid cultivars, Nco310, Q165 and R570. The solid grey histogram is the same histogram as shown in Figure 4., the histogram shown as a dark grey outline is the second histogram generated. Duplicate histograms were generated for all genotypes except Comus.
